# Supplementary material for: An Evolutionarily Conserved Coreceptor Gene Is Essential for CLAVATA Signaling in Marchantia polymorpha
Source: Front Plant Sci. 2021 Apr 13;12:657548. doi: 10.3389/fpls.2021.657548 (PMC8076897; doi:10.3389/fpls.2021.657548)
Supplement: Supplementary file 1 [file Presentation_1.PPTX]

## Slide 1
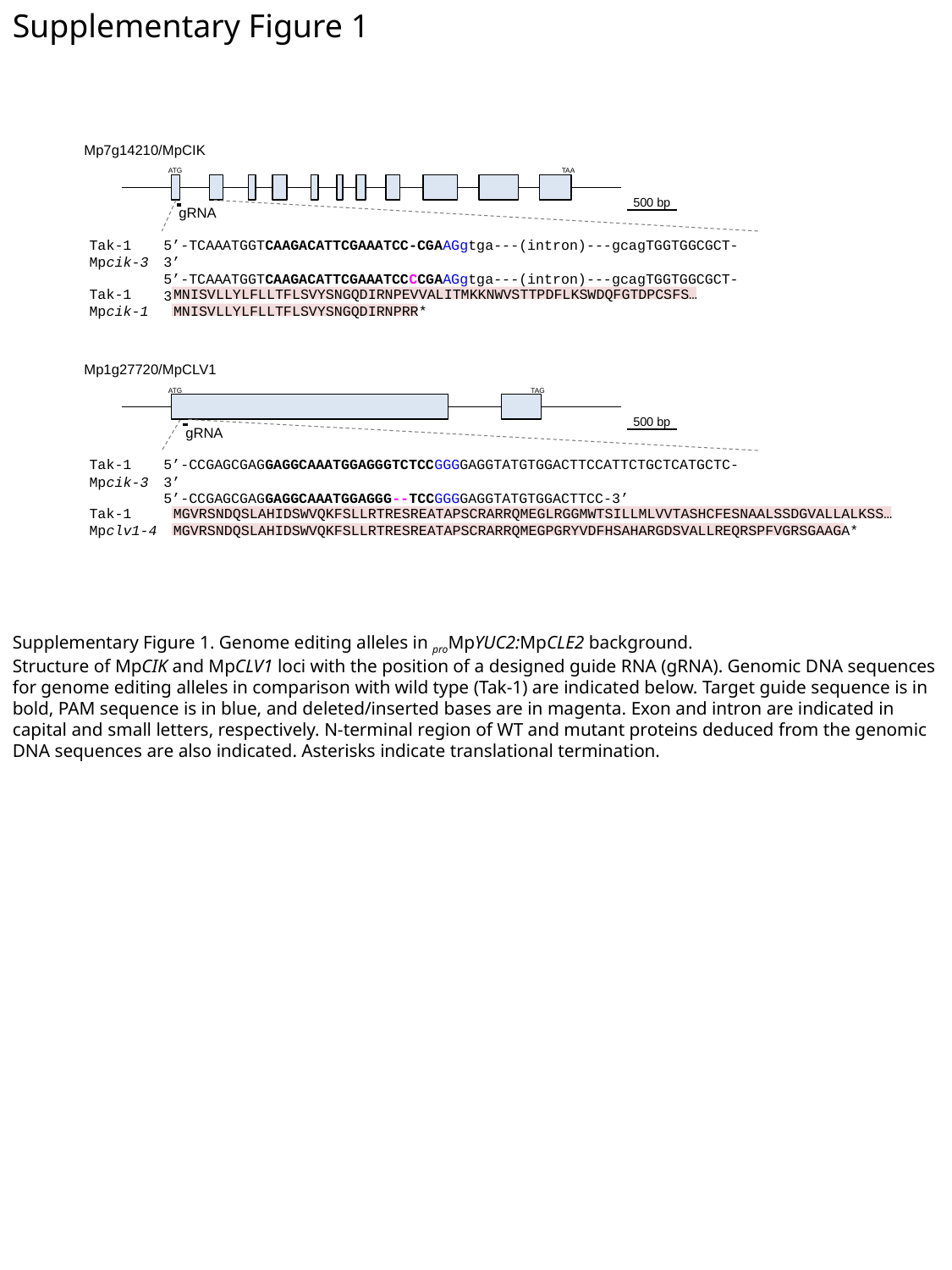

Supplementary Figure 1
Mp7g14210/MpCIK
ATG
TAA
500 bp
gRNA
Tak-1
Mpcik-3
5’-TCAAATGGTCAAGACATTCGAAATCC-CGAAGgtga---(intron)---gcagTGGTGGCGCT-3’
5’-TCAAATGGTCAAGACATTCGAAATCCCCGAAGgtga---(intron)---gcagTGGTGGCGCT-3’
Tak-1
Mpcik-1
MNISVLLYLFLLTFLSVYSNGQDIRNPEVVALITMKKNWVSTTPDFLKSWDQFGTDPCSFS…
MNISVLLYLFLLTFLSVYSNGQDIRNPRR*
Mp1g27720/MpCLV1
ATG
TAG
500 bp
gRNA
Tak-1
Mpcik-3
5’-CCGAGCGAGGAGGCAAATGGAGGGTCTCCGGGGAGGTATGTGGACTTCCATTCTGCTCATGCTC-3’
5’-CCGAGCGAGGAGGCAAATGGAGGG--TCCGGGGAGGTATGTGGACTTCC-3’
Tak-1
Mpclv1-4
MGVRSNDQSLAHIDSWVQKFSLLRTRESREATAPSCRARRQMEGLRGGMWTSILLMLVVTASHCFESNAALSSDGVALLALKSS…
MGVRSNDQSLAHIDSWVQKFSLLRTRESREATAPSCRARRQMEGPGRYVDFHSAHARGDSVALLREQRSPFVGRSGAAGA*
Supplementary Figure 1. Genome editing alleles in proMpYUC2:MpCLE2 background.
Structure of MpCIK and MpCLV1 loci with the position of a designed guide RNA (gRNA). Genomic DNA sequences for genome editing alleles in comparison with wild type (Tak-1) are indicated below. Target guide sequence is in bold, PAM sequence is in blue, and deleted/inserted bases are in magenta. Exon and intron are indicated in capital and small letters, respectively. N-terminal region of WT and mutant proteins deduced from the genomic DNA sequences are also indicated. Asterisks indicate translational termination.
